# Prevalence of Five Clinically Relevant Genital Microorganisms Among Women in South America, 2014–2024: A Systematic Review and Meta-Analysis

**DOI:** 10.3390/pathogens15090888

**Published:** 2026-08-24

**Authors:** Verónica Gabriela Osorio Pozo, María Gabriella Rodríguez-Marcano, Carlos Bastidas-Caldes, Ana M. Martínez-Pérez, Manuel Calvopiña

**Affiliations:** 1 School of Medicine, Universidad de las Américas, Quito 170124, Ecuador; 2 PhD Programme in Epidemiology and Public Health, International Doctoral School, Universidad Rey Juan Carlos, 28922 Madrid, Spain; 3 School of Medicine, Universidad Espíritu Santo, Samborondón 0901952, Ecuador; 4 Instituto Nacional de Biodiversidad (INABIO), Quito 170515, Ecuador; 5 Department of Communication and Sociology, Faculty of Communication Sciences, Universidad Rey Juan Carlos, 28942 Madrid, Spain; 6 One Health Research Group, School of Medicine, Universidad de las Américas, Quito 170124, Ecuador

**Keywords:** genital tract infections, vaginal discharge, vaginal dysbiosis, South America, systematic review and meta-analysis

## Abstract

**Background:** Genital tract infections and dysbiosis-related conditions contribute substantially to women’s sexual and reproductive morbidity. In South America, evidence on clinically relevant genital microorganisms remains fragmented across countries, populations, and diagnostic approaches. This systematic review and meta-analysis synthesized the prevalence of *Chlamydia trachomatis*, *Neisseria gonorrhoeae*, *Trichomonas vaginalis*, *Gardnerella vaginalis*, and *Candida* spp. among women in South America. **Methods:** We searched PubMed/MEDLINE, Web of Science, Cochrane Library, and SciELO for studies published from 1 January 2014 to 29 February 2024. Eligible studies reported prevalence data for at least one target microorganism in female populations from South American countries. Two reviewers independently screened studies, extracted data, and assessed methodological quality using Joanna Briggs Institute criteria for prevalence studies. Crude prevalence and random-effects pooled prevalence estimates with 95% confidence intervals were calculated. The review protocol was registered in PROSPERO (CRD420261324060). **Results:** Of 629 records identified, 51 studies from eight South American countries and French Guiana met the eligibility criteria for the systematic review, and 46 contributed to the main pooled prevalence meta-analysis. Substantial between-study heterogeneity was observed across all pathogen-specific analyses. Pooled prevalence was highest for *G. vaginalis* (28%), followed by *Candida* spp. (12%), *C. trachomatis* (7%), *T. vaginalis* (6%), and *N. gonorrhoeae* (3.2%). **Conclusions:** This review highlights the heterogeneous burden of clinically relevant genital microorganisms among women in South America and supports standardized diagnostic approaches, clearer phenotypic definitions, and stronger surveillance across underrepresented regional settings.

## 1. Introduction

Sexually transmitted infections (STIs) continue to represent a substantial public health challenge worldwide because of their high frequency, their often asymptomatic presentation, and their impact on sexual and reproductive health. According to the World Health Organization, curable STIs remain highly prevalent and contribute to important adverse outcomes, including pelvic inflammatory disease, infertility, complications during pregnancy, neonatal morbidity, and increased susceptibility to other infections. Global estimates also indicate that *C. trachomatis*, *N. gonorrhoeae*, and *T. vaginalis* continue to affect millions of women, highlighting the need for robust epidemiological surveillance and reliable diagnostic approaches [1,2,3].

Among women, lower genital tract infections and dysbiosis-related conditions are common reasons for seeking medical care and represent an important component of sexual and reproductive morbidity. These conditions do not correspond to a single clinical entity; rather, they may reflect a broad range of infectious and dysbiotic processes with different biological behaviors, clinical implications, and transmission patterns. In this context, *C. trachomatis*, *N. gonorrhoeae*, and *T. vaginalis* are recognized sexually transmitted pathogens, whereas *G. vaginalis* and *Candida* spp. are commonly detected in bacterial vaginosis and vulvovaginal candidosis, respectively. Although these microorganisms differ in their pathophysiology and in their relationship to specific genital symptoms, all of them are clinically relevant in the broader evaluation of women’s lower genital tract and reproductive health [3,4,5,6,7].

The relevance of these microorganisms extends beyond symptom-based care. *G. vaginalis* has been closely linked to the pathogenesis of bacterial vaginosis and to alterations in the vaginal microbiota, which in turn have been associated with inflammation, increased susceptibility to sexually transmitted infections, and unfavorable reproductive outcomes. Likewise, vulvovaginal candidosis remains one of the most frequent causes of vulvovaginal discomfort in women of reproductive age. From an epidemiological perspective, the vaginal microbiome plays an important role in shaping the local environment in which both dysbiosis-related conditions and sexually transmitted pathogens may emerge, persist, or coexist. Evaluating these microorganisms together may therefore offer a more clinically meaningful and epidemiologically comprehensive picture of genital infections among women [4,5,6,7].

In Latin America, and particularly in South America, this issue is further influenced by structural and social determinants that affect women’s sexual and reproductive health. Unequal access to health services, delayed diagnosis, barriers to timely treatment, and vulnerability among adolescents and other underserved populations may all contribute to the persistence and underrecognition of genital infections. Regional evidence has shown that prevalence estimates vary widely across countries and populations, while public health organizations have repeatedly emphasized the need for gender-sensitive and equity-oriented responses in sexual and reproductive health. Despite this, much of the available literature in the region has focused on selected pathogens, specific age groups, or particular high-risk populations, rather than providing an integrated overview of clinically relevant genital microorganisms across women in South America [8,9,10,11].

In addition, the evidence available from South America remains fragmented. Studies differ considerably in terms of population characteristics, laboratory methods, sampling strategies, and the specific microorganisms investigated. This heterogeneity makes it difficult to compare results across settings and to build a clear regional picture of the prevalence of key genital microorganisms among women. A more integrated synthesis is therefore needed, particularly one that does not rely on a purely syndromic framework, but instead examines the distribution of individual microorganisms of clinical and epidemiological importance [8,12,13].

Therefore, the aim of this systematic review and meta-analysis was to estimate the prevalence of five clinically relevant genital microorganisms (i.e., *G. vaginalis*, *Candida* spp., *C. trachomatis*, *T. vaginalis*, and *N. gonorrhoeae*) among women in South America between 2014 and 2024. By focusing on the prevalence of these specific microorganisms across female populations from the region, this study seeks to provide a regional synthesis to support epidemiological surveillance, diagnostic strategies, and sexual and reproductive health policy in South America.

## 2. Materials and Methods

### 2.1. Area of Study

South America is the southern subregion of the American continent. The subcontinent comprises 12 countries—Argentina, Bolivia, Brazil, Chile, Colombia, Ecuador, Guyana, Paraguay, Peru, Suriname, Uruguay, and Venezuela—as well as one overseas territory, French Guiana. South America is characterized by marked ecological and demographic heterogeneity. While most countries lie within the tropical belt, a substantial portion of the Southern Cone extends into mid-latitude regions. These geographic, climatic, and socio-economic contrasts contribute to substantial variation in sexual health determinants, diagnostic capacity, and epidemiological surveillance systems across the region, making South America a particularly relevant setting for comparative analyses of five clinically relevant microorganisms involved in women’s lower genital tract infections.

### 2.2. Eligibility Criteria

Studies were considered eligible if they were published between 1 January 2014 and 29 February 2024, were conducted in South American countries or French Guiana, and reported original quantitative detection data for at least one of the five target microorganisms in female populations. No language restrictions were applied. The target microorganisms were *C. trachomatis*, *N. gonorrhoeae*, *T. vaginalis*, *G. vaginalis*, and *Candida* spp. Eligible quantitative data were operationally defined as a clearly specified number of women tested and a corresponding number of positive cases, or sufficient information to derive these numerator and denominator values reliably. For studies based on clinical, convenience, screening-based, or otherwise-selected samples, the resulting proportion was interpreted as positivity within the sampled population rather than as a population-representative prevalence estimate. No minimum sample-size threshold was applied. Reports presenting only percentages without a recoverable numerator or denominator, data restricted to preselected positive specimens, or results that could not be separated for the eligible female population were excluded.

Observational and interventional study designs were eligible, including cross-sectional, cohort, and randomized studies, provided that the diagnostic methodology was clearly described. For studies including mixed-sex populations, only female-specific data were extracted. For interventional studies, only baseline or otherwise-prevalence-comparable data collected before the intervention were considered. Studies were excluded if they focused exclusively on male populations, were conducted outside the predefined geographic area, or corresponded to reviews, conference abstracts, editorials, protocols, case reports, or letters without original quantitative data. Additional exclusion criteria included insufficient description of diagnostic techniques, absence of extractable pathogen-specific prevalence data, and unavailability of the full-text article after reasonable retrieval efforts. The full eligibility criteria and associated screening specifications are detailed in Table S1.

### 2.3. Protocol and Registration

**Figure 1:**
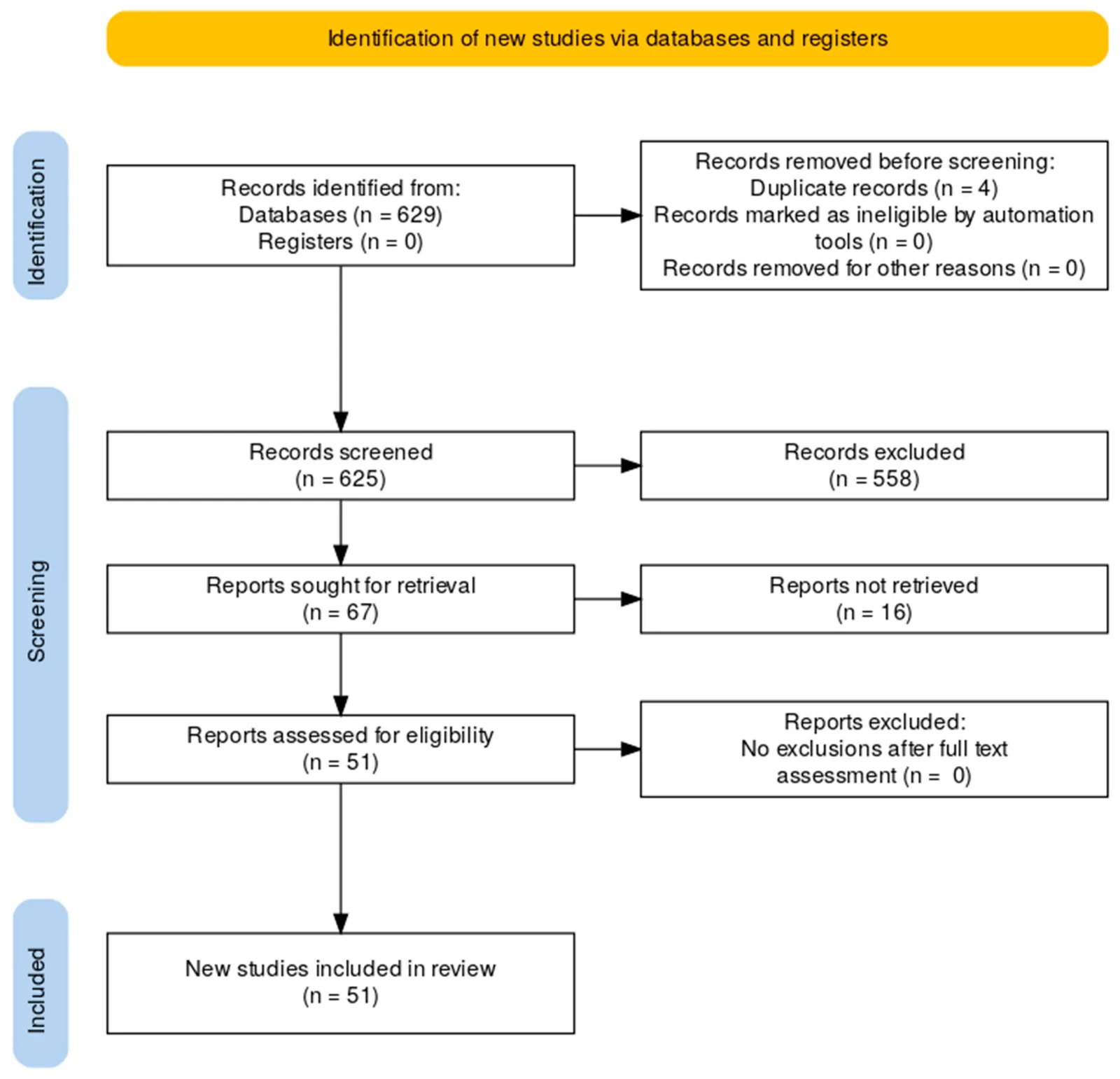
PRISMA 2020 flow diagram showing study identification, screening, retrieval, and inclusion in the systematic review. All 51 retrieved full-text reports met the eligibility criteria for the systematic review; 46 were subsequently included in the main pooled prevalence meta-analysis, whereas 5 were retained for qualitative synthesis only because their designs or sampling frameworks did not support directly comparable prevalence inference. Available bibliographic details and retrieval information for the 16 reports not obtained are provided in Table S9.

This systematic review and meta-analysis was conducted in accordance with the Preferred Reporting Items for Systematic Reviews and Meta-Analyses (PRISMA) 2020 guidelines [14]. The completed PRISMA 2020 checklist is provided as Checklist S1. The study protocol was registered in the PROSPERO International Prospective Register of Systematic Reviews under registration number CRD420261324060 on 5 March 2026 and is available at https://www.crd.york.ac.uk/PROSPERO/view/CRD420261324060. The registry record was subsequently amended, with the most recent edit completed on 21 June 2026, to reflect the major post-registration protocol changes described below. The study selection process is summarized in Figure 1, which presents the PRISMA 2020 flow diagram detailing the identification, screening, eligibility assessment, and final inclusion of studies.

Following registration, several protocol amendments were made to align the review with the broader regional evidence identified during study selection and data synthesis. First, the eligible population was expanded from women presenting with abnormal vaginal discharge to female populations in South America more generally, including symptomatic, asymptomatic, prenatal-care, screening-based, routine-care, and selected or high-risk populations. Second, the information sources and database-specific search strategies were updated to include PubMed/MEDLINE, Web of Science, the Cochrane Library, and SciELO, with explicit date limits from 1 January 2014 to 29 February 2024, no language restrictions, and backward citation searching. Third, the methodological appraisal procedures were refined by distinguishing the JBI-based overall risk-of-bias assessment from study suitability for inclusion in the primary pooled prevalence meta-analysis. The reporting-bias assessment was restricted to pathogen-specific analyses with at least 10 studies, certainty of evidence was evaluated using an adapted GRADE-style approach, and the planned secondary analyses were revised to include exploratory subgroup analyses by diagnostic method and population type, together with descriptive cross-study analyses of reported demographic and behavioral covariates. These amendments did not change the five prespecified target microorganisms.

### 2.4. Information Sources and Search Strategy

A systematic literature search was conducted in PubMed/MEDLINE, Web of Science, the Cochrane Library, and SciELO, with the final search performed on 29 February 2024. Across databases, the core search framework combined terms for the five target microorganisms—*Neisseria gonorrhoeae*, *Chlamydia trachomatis*, *Gardnerella vaginalis*, *Candida* spp., and *Trichomonas vaginalis*—using the Boolean operator OR. This pathogen block was combined with geographic terms comprising “South America,” Argentina, Bolivia, Brazil, Chile, Colombia, Ecuador, Guyana, Paraguay, Peru, Suriname, Uruguay, Venezuela, and French Guiana. PubMed/MEDLINE searches incorporated MeSH terms and title/abstract fields, whereas Web of Science, the Cochrane Library, and SciELO used database-adapted free-text syntax and field specifications. No language restrictions were applied, and the searches were restricted to studies published between 1 January 2014 and 29 February 2024. The complete database-specific search strategies are provided in Table S1. Backward citation searching of the reference lists of eligible studies and relevant reviews was additionally conducted to identify potentially eligible records not retrieved through database searching.

### 2.5. Study Selection and Data Extraction

Two reviewers (VGOP and MGR) independently screened titles and abstracts, followed by full-text assessment of potentially eligible articles. Disagreements were resolved through discussion and consensus, with the involvement of a third reviewer when necessary (CB). Data extraction was performed independently by two reviewers using a standardized matrix developed for the included studies, and discrepancies were resolved through discussion and consensus. The matrix captured bibliographic information, country of origin, population characteristics, and key epidemiological variables, including study population type, reported demographic and behavioral covariates, diagnostic methods, sample size, number of confirmed cases, and reported prevalence estimates for each evaluated microorganism (Table S2).

### 2.6. Risk of Bias and Methodological Quality Assessment

Risk of bias was assessed independently by two reviewers using a structured critical appraisal approach based on the Joanna Briggs Institute (JBI) checklist for prevalence studies. For cross-sectional studies, appraisal covered the appropriateness of the sampling frame, participant recruitment, adequacy of sample size, description of study subjects and setting, coverage of the identified sample, validity of condition identification methods, standardization and reliability of outcome measurement, appropriateness of statistical analysis, and management of response or participation issues. For non-cross-sectional studies retained in the review, equivalent prevalence-oriented domains were applied to evaluate their suitability for prevalence synthesis. After item-level appraisal, each study was assigned an overall judgment of low, moderate, or high risk of bias for prevalence estimation using predefined decision rules. JBI items 1 and 2, addressing the appropriateness of the sampling frame and participant sampling or recruitment, were treated as critical domains because failures in these areas directly affect the representativeness of prevalence estimates. Studies were classified as low risk when neither critical domain was rated ‘No’, no more than one of the nine domains was unmet, and at least six domains were rated ‘Yes’. Moderate risk was assigned when one critical domain was rated ‘No’, when two or three domains were rated ‘No’ overall, or when fewer than six domains could be clearly rated ‘Yes’ because of accumulated ‘Unclear’ responses, provided that the criteria for high risk were not met. Studies were classified as high risk when both critical domains were rated ‘No’ or when four or more domains were rated ‘No’. ‘Unclear’ responses were not automatically counted as unmet domains but reduced confidence in the overall judgment. Separately, studies were evaluated for eligibility for the main pooled prevalence meta-analysis. Studies with highly structured sampling frameworks, lesion- or cytology-based selection, longitudinal follow-up designs not directly comparable with point-prevalence estimation, or microbiome-oriented designs that did not provide directly comparable pathogen prevalence measures were retained for qualitative synthesis but excluded from the primary pooled analyses. Disagreements were resolved through discussion and consensus, with consultation of a third reviewer when necessary. Full study-level JBI judgments, overall risk-of-bias classifications, decisions regarding inclusion in the main pooled prevalence meta-analysis, and standardized study-specific rationales for excluding the five non-comparable studies from quantitative pooling are presented in Table S3.

### 2.7. Statistical Analysis

The primary analysis consisted of pathogen-specific meta-analyses of prevalence proportions. Pooled prevalence estimates and corresponding 95% confidence intervals (95% CIs) were calculated using the metaprop function in the meta package in R version 4.1.3 (R Foundation for Statistical Computing, Vienna, Austria), applying a logit transformation of proportions (PLOGIT), inverse-variance weighting, and the DerSimonian–Laird estimator for between-study variance (τ^2^). Both common-effect and random-effects models were computed, but random-effects estimates were considered the primary summary measures given the expected clinical and methodological diversity across studies. For pathogen-specific meta-analyses containing at least one zero-event study, a continuity correction of 0.5 was applied to all contributing studies using the method.incr = “all” option. This conventional correction was selected because it is compatible with the logit-transformed prevalence framework, allows zero-event studies to remain in the synthesis, and applies the correction consistently across studies within the same meta-analysis. Hartung–Knapp adjustment and prediction intervals were not used. Pooled estimates were back-transformed to the prevalence scale for presentation. Statistical heterogeneity was quantified using the *I*^2^ statistic, with values >75% interpreted as indicating considerable between-study heterogeneity. Because prevalence meta-analyses are sensitive to sparse data, unequal study sizes, and between-study variability, pooled estimates were interpreted cautiously as regional summary measures rather than fixed epidemiological parameters. Forest plots were generated for each microorganism, and crude prevalence estimates based on observed positive cases over the total number tested (*n*/*N*) were additionally calculated to facilitate comparison between directly aggregated data and model-based pooled estimates.

**Figure 2:**
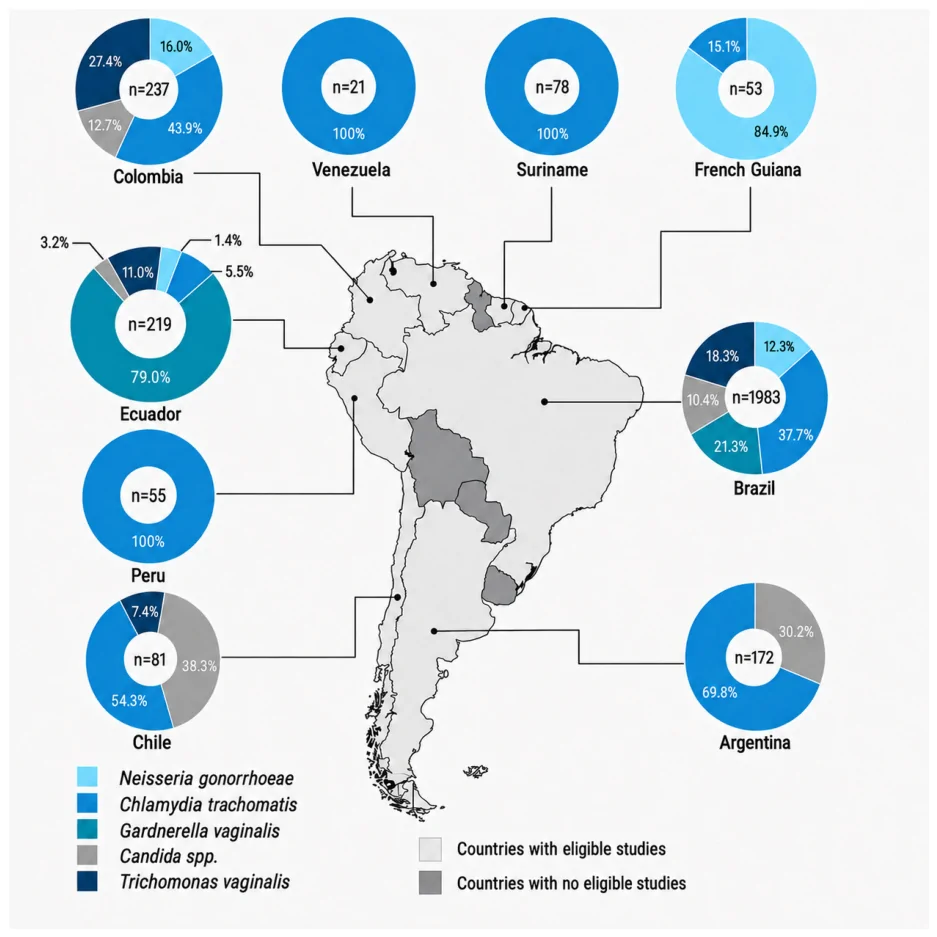
Geographic evidence map and country-level distribution of reported detections of five clinically relevant genital microorganisms among women in South America (2014–2024). Countries shaded in light gray indicate the presence of at least one eligible study reporting data for one or more of the investigated microorganisms, whereas countries shaded in dark gray indicate the absence of eligible data (Paraguay, Bolivia, and Uruguay). Donut charts illustrate the relative distribution of microorganism-positive detections reported within each country. Percentages represent the proportion of all reported positive detections in each country that were attributed to *C. trachomatis*, *N. gonorrhoeae*, *T. vaginalis*, *G. vaginalis*, and *Candida* spp., with the total number of reported positive detections per country (n) shown at the center of each chart. Microorganisms with no country-specific reported detections are not displayed. These proportions summarize the composition of reported detections across included studies and should not be interpreted as country-specific prevalence estimates or as equivalent to the pooled prevalence estimates presented in the meta-analysis.

The R code used for the main pathogen-specific meta-analyses, subgroup analyses, and funnel plots assessing reporting bias is provided in the repository indicated in the Data Availability Statement. The geographic evidence map and country-level graphical representation shown in Figure 2 were generated using QGIS (QGIS Geographic Information System, QGIS Association) and are not included in the analytical R code deposited in the repository.

Assessment of reporting bias and small-study effects was restricted to pathogen-specific meta-analyses that included at least 10 studies eligible for the main pooled prevalence synthesis. Funnel plots were visually inspected, and Egger regression tests were performed using logit-transformed prevalence estimates. A two-sided *p* value < 0.05 was considered indicative of funnel plot asymmetry. Because asymmetry in prevalence meta-analyses may arise from substantial heterogeneity, sparse data, unequal study sizes, or differences in population and study design, the findings were interpreted cautiously and were not considered evidence of publication bias in isolation.

Certainty of evidence for each pathogen-specific prevalence outcome was assessed using an adapted GRADE-style domain-based approach for prevalence studies. Judgments considered study risk of bias, inconsistency, indirectness, imprecision, and reporting bias or small-study effects, where assessable. Overall certainty was classified as high, moderate, low, or very low. Detailed certainty judgments are presented in Table S8.

As a secondary component, exploratory subgroup meta-analyses were performed to assess whether selected study-level characteristics could account for part of the observed between-study heterogeneity when a sufficient number of studies and comparable subgroup classifications were available. Studies were initially stratified according to diagnostic method and the original harmonized population-type classification presented in Table S4. These analyses were retained as the original exploratory subgroup analyses. In response to the clinical and geographic heterogeneity observed across the included studies, an additional set of post hoc exploratory subgroup analyses was subsequently conducted using three broader population-setting categories: selected/high-risk, urban general, and remote/rural populations. These additional analyses were intended to examine whether prevalence patterns differed according to a combined clinical-risk and geographic-setting framework and are presented separately in Figures S12–S16.

The selected/high-risk category included populations recruited on the basis of specific behavioral, clinical, or social characteristics associated with increased exposure or selective healthcare attendance, such as female sex workers, women living with HIV, homeless populations, or STI-oriented clinical groups. The urban general category included broader urban community, routine-care, antenatal-care, screening, or university populations without an explicit high-risk selection criterion. The remote/rural category included indigenous, geographically isolated, rural, or remote-community populations. Because some populations could plausibly overlap more than one category, classification was based on the principal sampling framework and recruitment setting reported by each study. The original population-type analyses and the additional three-category population-setting analyses should therefore be interpreted as complementary rather than interchangeable because they used different harmonization frameworks and were designed to address related but distinct aspects of population heterogeneity. Subgroup meta-analyses were performed using the same metaprop framework as the main analyses, including logit-transformed proportions, inverse-variance weighting, and DerSimonian–Laird estimation of between-study variance. For subgroup analyses containing at least one zero-event study, a continuity correction of 0.5 was applied to all contributing studies within the corresponding analysis using the method.incr = “all” option, and random-effects confidence intervals were calculated using the classic method. For each subgroup analysis, pooled prevalence estimates with 95% CIs were calculated under both common-effect and random-effects models. Formal differences between subgroups were evaluated using the between-subgroup Cochran’s Q statistic, reported as a chi-square test with the corresponding degrees of freedom and p value. Because substantial clinical and methodological heterogeneity was anticipated, subgroup-difference tests from the random-effects models were prioritized for interpretation, while common-effect results were retained in the supplementary forest plots for completeness. Because these subgroup analyses were based on observational studies with substantial clinical and methodological variability, they were considered exploratory and interpreted cautiously.

As a tertiary and strictly exploratory component, reported demographic and behavioral covariates were reviewed to identify whether any recurrent cross-study patterns could be described across pathogen-specific datasets. Study-level variables were harmonized into binary categories when feasible (Table S5). Descriptive cross-study comparisons were then conducted across pathogen–covariate pairs. Where the available reporting structure allowed, exploratory two-by-two comparisons and odds ratios (ORs) with 95% confidence intervals were estimated; however, these comparisons were not based on a standardized analytical framework across studies and should be interpreted solely as descriptive and hypothesis-generating. To reduce overinterpretation, *p* values were additionally adjusted for multiple testing using the Benjamini–Hochberg procedure (Table S6).

The extracted study-level dataset underlying the main prevalence meta-analyses, subgroup analyses, and exploratory cross-study comparisons is provided as Data S1.

## 3. Results

### 3.1. Study Selection

A total of 629 records were identified through database searching. After duplicate removal (*n* = 4), 625 records were screened, of which 558 were excluded at title and abstract review. Sixty-seven reports were sought for retrieval. Despite reasonable retrieval efforts, the complete reports could not be obtained for 16 records; their available bibliographic details and the retrieval approaches undertaken are summarized in Table S9. The remaining 51 full-text articles met the eligibility criteria for the systematic review [15,16,17,18,19,20,21,22,23,24,25,26,27,28,29,30,31,32,33,34,35,36,37,38,39,40,41,42,43,44,45,46,47,48,49,50,51,52,53,54,55,56,57,58,59,60,61,62,63,64,65]. Of these, 46 were considered suitable for inclusion in the main pooled prevalence meta-analysis, whereas five were retained for qualitative synthesis only because their design or sampling framework did not yield prevalence estimates directly comparable with the primary point-prevalence synthesis: Amorim et al. (2017) [21], Costa-Lira et al. (2017) [58], Hernández-Buelvas et al. (2021) [42], Mosmann et al. (2021) [45], and Witkin et al. (2021) [60]. These exclusions from the main pooled analysis were based on methodological comparability criteria for prevalence synthesis and should not be interpreted as reflecting lower relevance to the review question (Figure 1).

### 3.2. Geographic Distribution of Included Studies and Reported Microorganism Detections

The final 51 studies included in this review were conducted across eight South American countries and French Guiana. Most were carried out in Brazil (31/51; 60.8%), followed by Colombia (5/51; 9.8%), Argentina (5/51; 9.8%), Chile (4/51; 7.8%), Ecuador (2/51; 3.9%), and one study each from Peru, Suriname, Venezuela, and French Guiana (1/51; 2.0% each). No eligible studies were identified from Paraguay, Bolivia, or Uruguay within the predefined time window. Figure 2 summarizes the composition of microorganism-positive detections reported by the included studies within each country. The donut charts display the proportion of all positive detections attributed to each of the five target microorganisms and are therefore influenced by the microorganisms investigated, the number and design of contributing studies, the populations sampled, and the diagnostic methods used in each country. Accordingly, differences in chart composition should not be interpreted as evidence of differences in population-level prevalence, national disease burden, or relative infection risk between countries. Figure 2 is intended solely as a descriptive visualization of the available reported detections and the geographical distribution of the evidence base.

### 3.3. Characteristics of Included Studies

Across the 51 included studies, cross-sectional designs predominated (*n* = 43, 84.3%), with only a small number of studies using other observational designs. Sample sizes varied substantially, ranging from approximately 100 to 35,886 participants. Most studies enrolled fewer than 500 women (*n* = 39, 76.5%), followed by studies including between 500 and 4999 participants (*n* = 11, 21.6%), while one study included at least 5000 participants (*n* = 1, 2.0%). Regarding study populations, 14 studies (27.5%) focused on women attending gynecologic or prenatal care services, six (11.8%) included symptomatic clinic attendees, and one (2.0%) specifically evaluated an indigenous population; the remaining studies (*n* = 30, 58.8%) assessed broader or mixed female populations. Diagnostic approaches and specimen types were also heterogeneous. Molecular methods predominated, being used in 47/51 studies (92.2%), most commonly PCR-based assays, whereas conventional techniques were less frequent and included culture in 6/51 studies (11.8%), microscopy in 3/51 (5.9%), Gram staining in 3/51 (5.9%), and clinical diagnosis in 1/51 (2.0%); several studies used more than one diagnostic approach. Vaginal or cervicovaginal samples were the most frequently used specimen type (22/51, 43.1%), followed by cervical or endocervical samples (13/51, 25.5%), mixed specimen types (10/51, 19.6%), urine samples (5/51, 9.8%), and genital specimens (1/51, 2.0%). This methodological heterogeneity may have contributed to the substantial between-study variability observed in the meta-analyses.

A comprehensive summary of study-level characteristics, including study design, sample size, population profile, specimen type, and diagnostic methods for the five evaluated pathogens, is presented in Table S2.

### 3.4. Methodological Appraisal and Study Suitability

Among the 51 included studies, 16 (31.4%) were classified as having low risk of bias, 29 (56.9%) as moderate risk, and 6 (11.8%) as high risk for prevalence estimation. The main methodological concerns were related to selective clinical recruitment, limited representativeness of the source population, restriction to highly specific subgroups, and sampling frameworks that were not directly comparable with prevalence-oriented cross-sectional designs. In contrast, the specimen type and general laboratory methodology were extractable for all 51 included studies. In the JBI appraisal, 49 studies (96.1%) were judged to have used valid methods for condition identification, whereas one study (2.0%) was rated ‘Unclear’ and one (2.0%) was rated ‘No’. Similarly, measurement was considered standardized and reliable across all participants in 49 studies (96.1%), while two studies (3.9%) were rated ‘No’. These findings indicate that diagnostic procedures were generally well described and commonly based on molecular methods, suggesting that between-study variability was driven more by differences in sampled populations and recruitment settings than by laboratory ascertainment alone.

For quantitative synthesis, 46 of the 51 studies (90.2%) were considered eligible for inclusion in the main pooled prevalence meta-analysis, whereas five studies (9.8%) were excluded because their design or sampling framework did not support directly comparable prevalence inference: Amorim et al. (2017) [21], Costa-Lira et al. (2017) [58], Hernández-Buelvas et al. (2021) [42], Mosmann et al. (2021) [45], and Witkin et al. (2021) [60]. The main reasons for exclusion included intentionally structured sampling according to lesion or cytology status, longitudinal or cohort-based designs not directly comparable with point-prevalence studies, and microbiome-oriented investigations that did not provide directly comparable pathogen prevalence estimates. Full study-level JBI judgments, overall risk-of-bias classifications, and decisions regarding inclusion in the main pooled prevalence meta-analysis are presented in Table S3.

### 3.5. Pooled Prevalence of Five Clinically Relevant Genital Microorganisms

**Figure 3:**
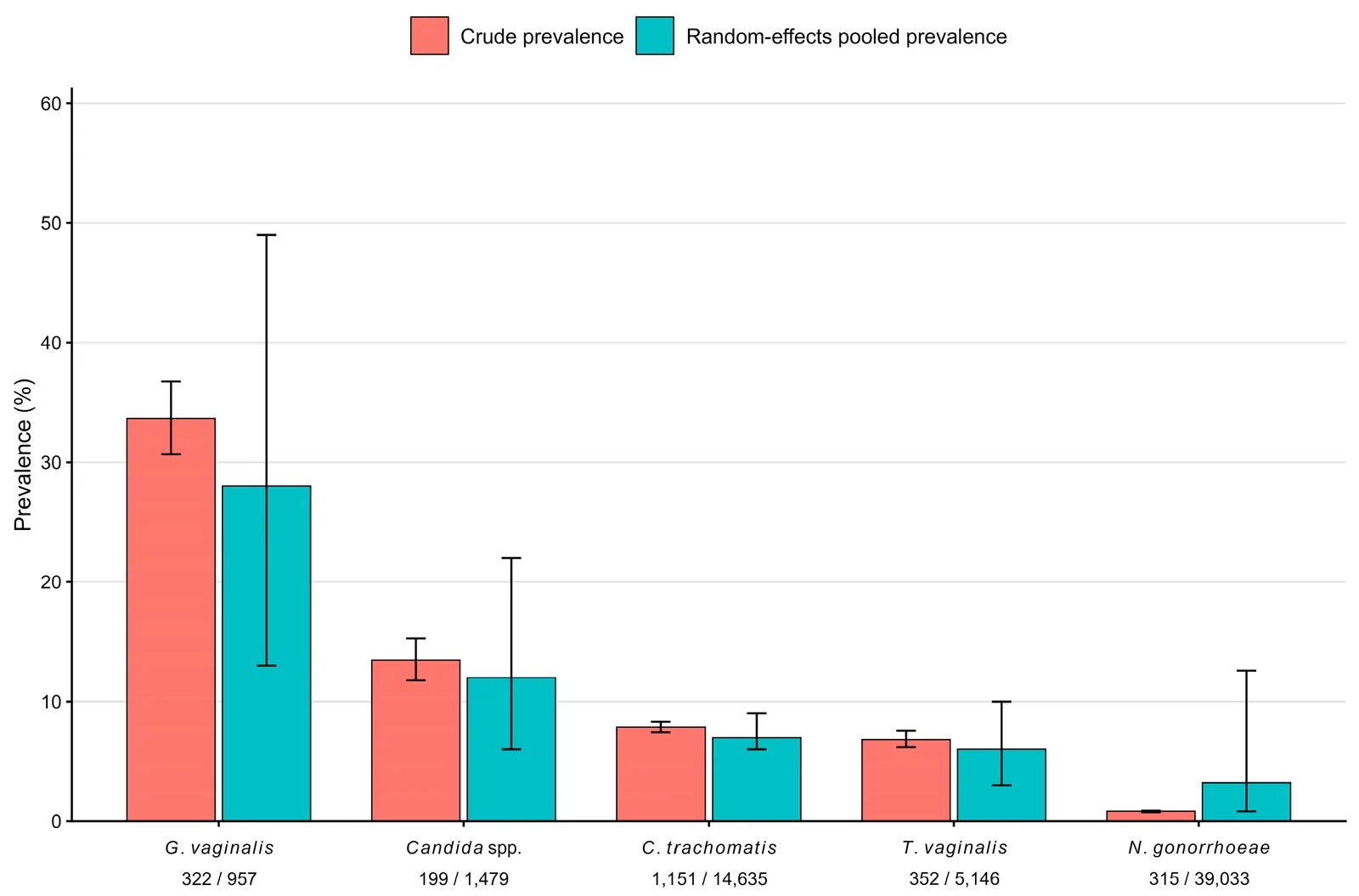
Crude prevalence and random-effects pooled prevalence estimates for five clinically relevant genital microorganisms among women in eight South American countries and French Guiana, 2014–2024. Bars represent the crude and pooled prevalence estimates, and vertical error bars indicate the corresponding 95% confidence intervals, not standard deviations or standard errors. Crude prevalence was calculated by directly aggregating the number of positive cases and the total number of women tested, with 95% confidence intervals based on the binomial distribution. Pooled prevalence and its corresponding 95% confidence interval were estimated using pathogen-specific random-effects meta-analysis models. Differences between crude and pooled estimates reflect study weighting, unequal study sizes, and between-study heterogeneity.

**Figure 4:**
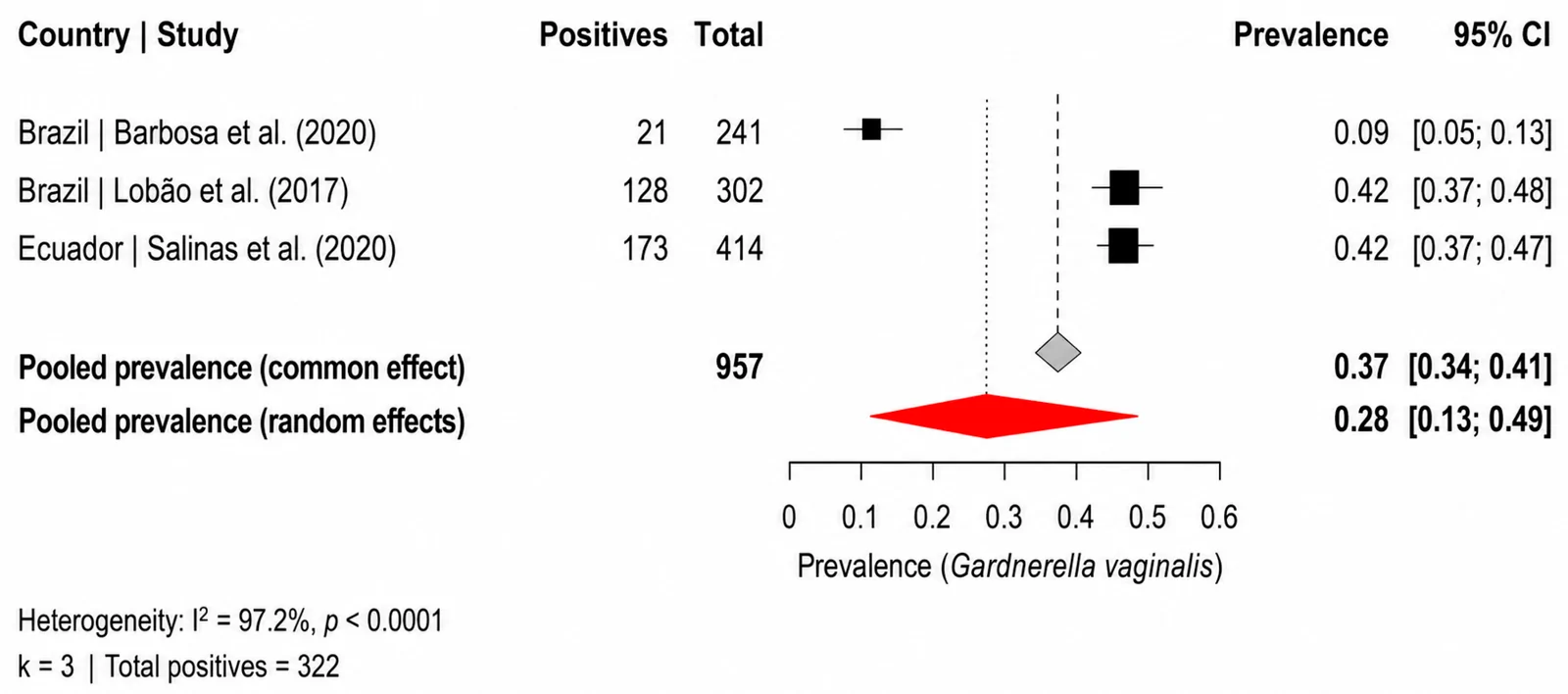
Forest plot of *G. vaginalis* prevalence across three studies conducted in South America between 2017 and 2020 [23,38,59]. The pooled prevalence was 37% (95% CI: 34–41%) under the common-effect model and 28% (95% CI: 13–49%) under the random-effects model, with considerable heterogeneity (*I*^2^ = 97.2%, *p* < 0.0001). Each horizontal line represents an individual study with its 95% confidence interval, the size of each square is proportional to the study weight, and the diamonds indicate the pooled estimates.

**Figure 5:**
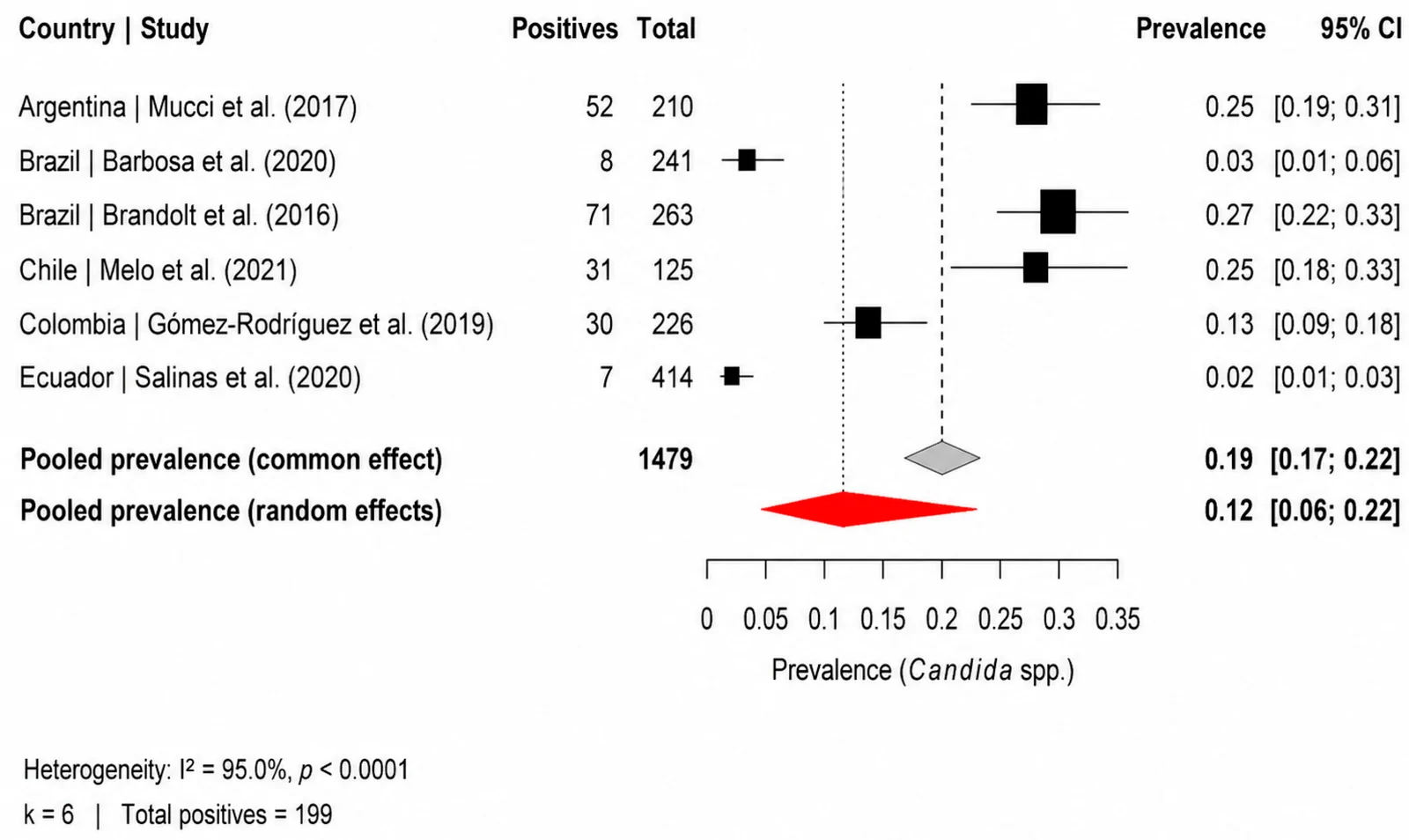
Forest plot of *Candida* spp. prevalence across six studies conducted in South America between 2016 and 2021 [16,23,41,43,46,59]. The pooled prevalence was 19% (95% CI: 17–22%) under the common-effect model and 12% (95% CI: 6–22%) under the random-effects model, with considerable heterogeneity (*I*^2^ = 95.0%, *p* < 0.0001). Each horizontal line represents an individual study with its 95% confidence interval, the size of each square is proportional to the study weight, and the diamonds indicate the pooled estimates.

**Figure 6:**
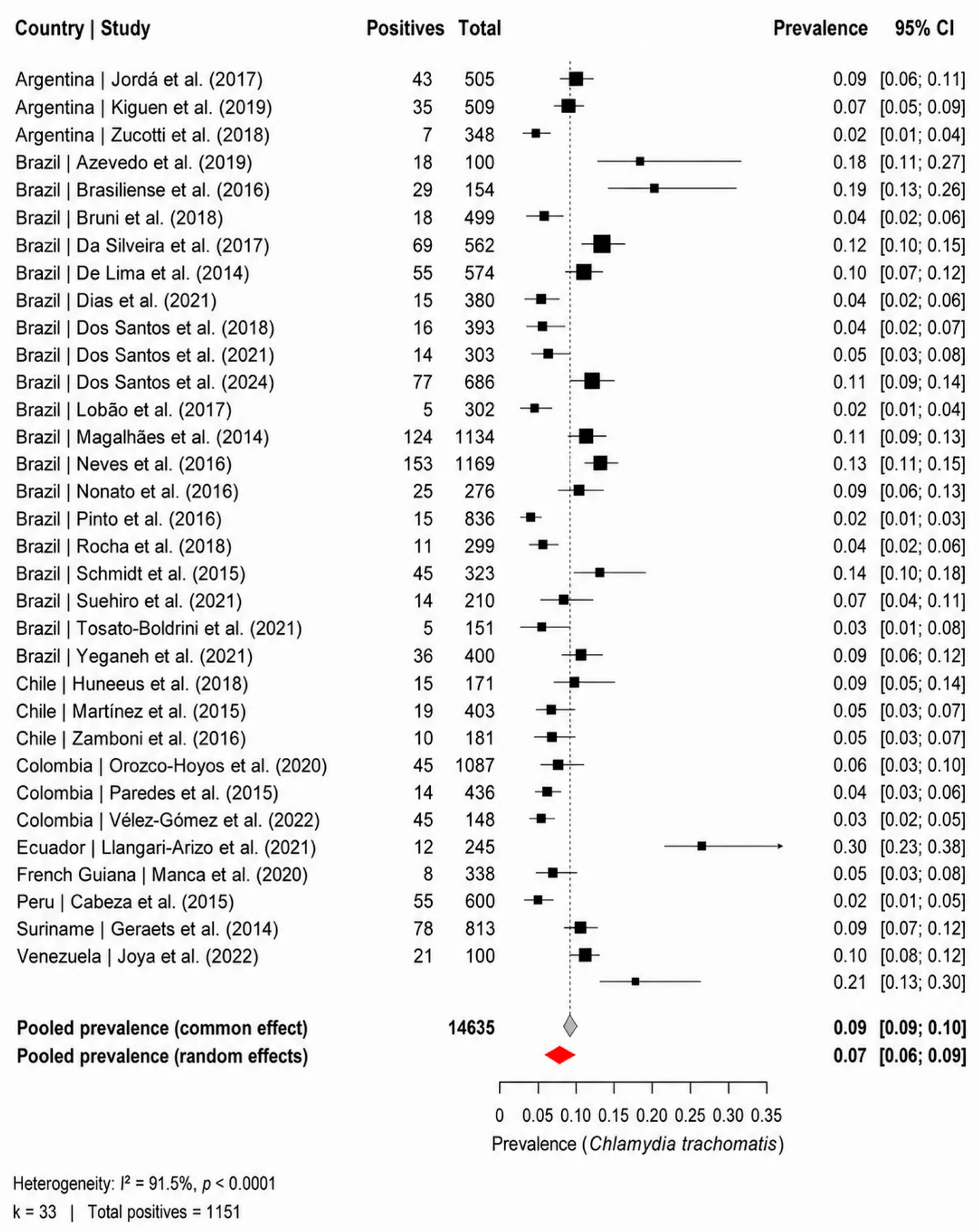
Forest plot of *C. trachomatis* prevalence across 33 studies conducted in South America between 2014 and 2024 [17,18,19,22,24,25,27,28,29,31,32,33,34,35,36,37,38,39,40,47,48,49,50,51,52,54,55,56,57,61,62,63,64]. The pooled prevalence was 9% (95% CI: 9–10%) under the common-effect model and 7% (95% CI: 6–9%) under the random-effects model, with considerable heterogeneity (*I*^2^ = 91.5%, *p* < 0.0001). Each horizontal line represents an individual study with its 95% confidence interval, the size of each square is proportional to the study weight, and the diamonds indicate the pooled estimates.

**Figure 7:**
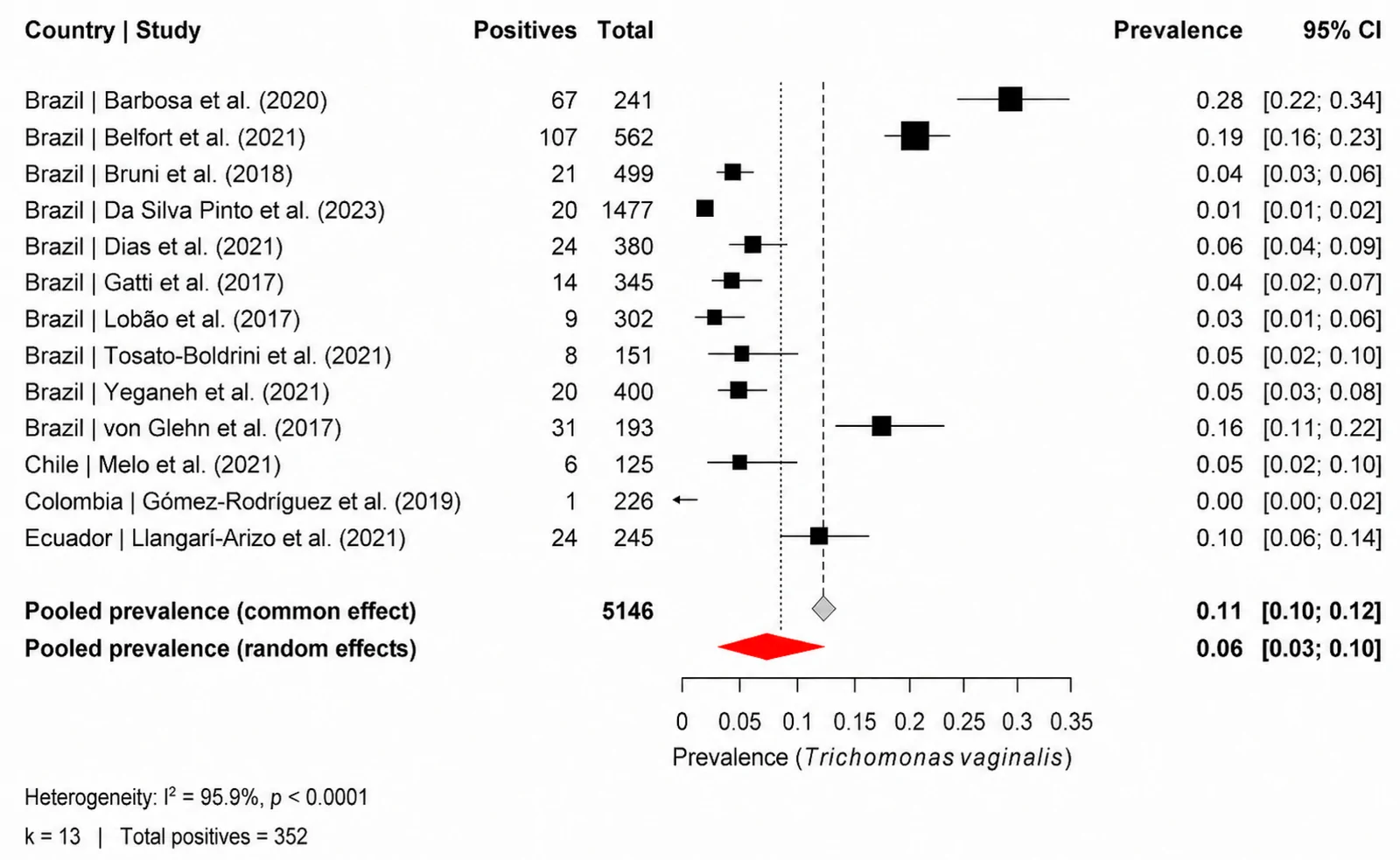
Forest plot of *T. vaginalis* prevalence across 13 studies conducted in South America between 2014 and 2023 [15,18,23,25,26,30,37,38,41,43,54,61,65]. The pooled prevalence was 11% (95% CI: 10–12%) under the common-effect model and 6% (95% CI: 3–10%) under the random-effects model, with considerable heterogeneity (*I*^2^ = 95.9%, *p* < 0.0001). Each horizontal line represents an individual study with its 95% confidence interval, the size of each square is proportional to the study weight, and the diamonds indicate the pooled estimates.

**Figure 8:**
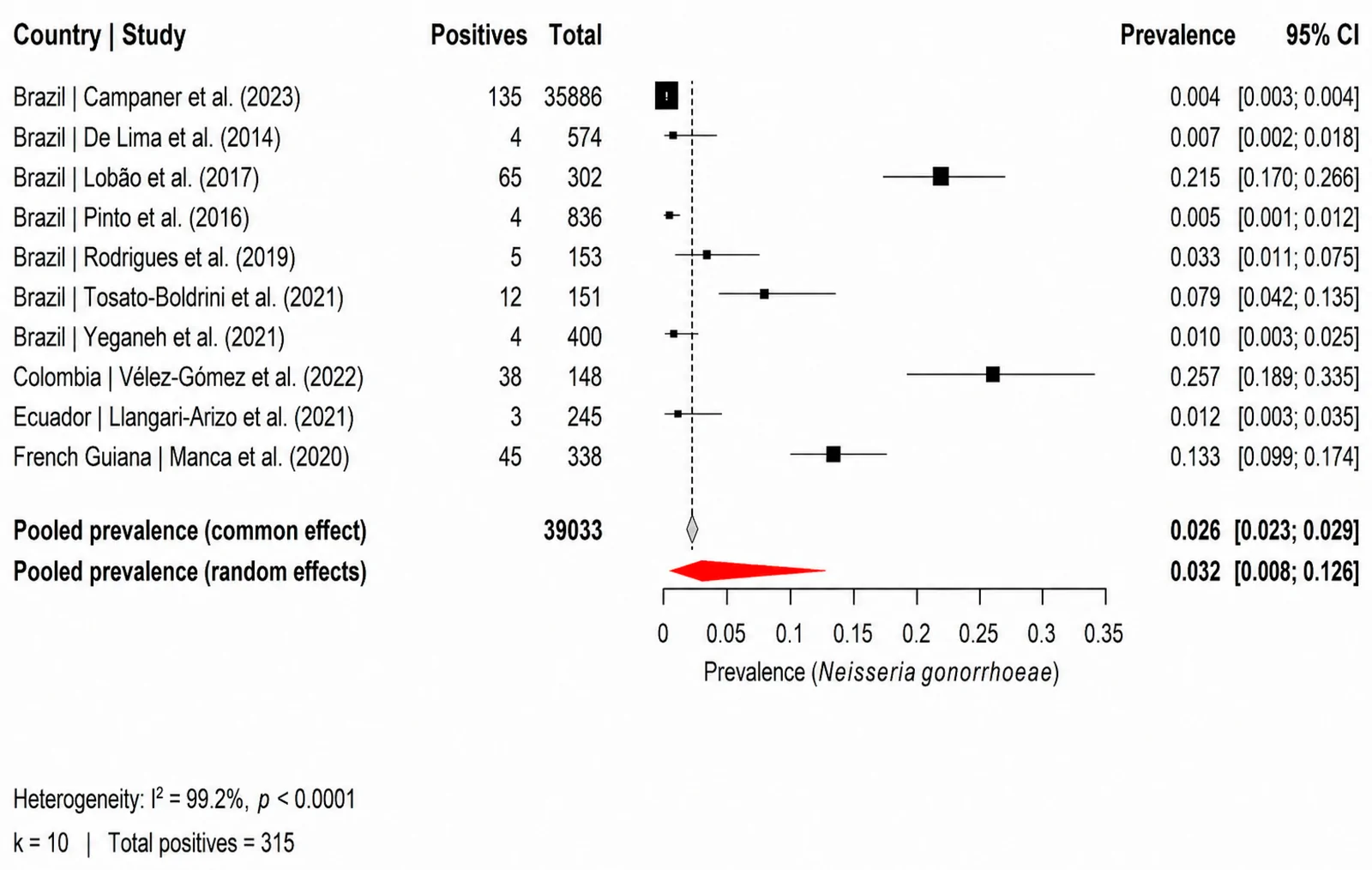
Forest plot of *N. gonorrhoeae* prevalence across 10 studies conducted in South America between 2014 and 2023 [20,24,37,38,40,48,53,54,61,64]. The pooled prevalence was 2.6% (95% CI: 2.3–2.9%) under the common-effect model and 3.2% (95% CI: 0.8–12.6%) under the random-effects model, with considerable heterogeneity (*I*^2^ = 99.2%, *p* < 0.0001). Each horizontal line represents an individual study with its 95% confidence interval, the size of each square is proportional to the study weight, and the diamonds indicate the pooled estimates.

Crude prevalence estimates and random-effects pooled prevalence estimates are presented in Table 1 and summarized in Figure 3, with detailed forest plots provided in Figure 4, Figure 5, Figure 6, Figure 7 and Figure 8. Pooled prevalence estimates shown in the pathogen-specific forest plots correspond to the primary quantitative synthesis restricted to studies considered eligible for the main pooled prevalence meta-analysis, as defined in Table S3. Detailed population characteristics for each study are reported in Table S2. The original harmonized population-type classification is presented in Table S4, with the corresponding diagnostic-method and population-type subgroup analyses shown in Figures S1–S8. Additional exploratory analyses using selected/high-risk, urban general, and remote/rural population-setting categories are presented separately in Figures S12–S16.

#### 3.5.1. *G. vaginalis*

Three studies reported *G. vaginalis*, with a crude prevalence of 33.65% (322/957). The pooled prevalence under the random-effects model was 28% (95% CI: 13–49%), with considerable heterogeneity across studies (*I*^2^ = 97.2%). The corresponding forest plot is shown in Figure 4.

#### 3.5.2. *Candida* spp.

Six studies reported *Candida* spp., with a crude prevalence of 13.46% (199/1479). The pooled prevalence under the random-effects model was 12% (95% CI: 6–22%), with considerable heterogeneity across studies (*I*^2^ = 95.0%). The corresponding forest plot is shown in Figure 5.

#### 3.5.3. *C. trachomatis*

Thirty-three studies reported *C. trachomatis*, with a crude prevalence of 7.86% (1151/14,635). The pooled prevalence under the random-effects model was 7% (95% CI: 6–9%), with considerable heterogeneity across studies (*I*^2^ = 91.5%). The corresponding forest plot is shown in Figure 6.

#### 3.5.4. *T. vaginalis*

Thirteen studies reported *T. vaginalis*, with a crude prevalence of 6.84% (352/5146). The pooled prevalence under the random-effects model was 6% (95% CI: 3–10%), with considerable heterogeneity across studies (*I*^2^ = 95.9%). The corresponding forest plot is shown in Figure 7.

#### 3.5.5. *N. gonorrhoeae*

Ten studies reported *N. gonorrhoeae*, with a crude prevalence of 0.81% (315/39,033). The pooled prevalence under the random-effects model was 3.2% (95% CI: 0.8–12.6%), with considerable heterogeneity across studies (*I*^2^ = 99.2%). The corresponding forest plot is shown in Figure 8.

### 3.6. Heterogeneity Across Studies

Across all five pathogen-specific meta-analyses, between-study heterogeneity was consistently considerable, with *I*^2^ values ranging from 91.5% to 99.2%. Specifically, heterogeneity was 97.2% for *G. vaginalis*, 95.0% for *Candida* spp., 91.5% for *C. trachomatis*, 95.9% for *T. vaginalis*, and 99.2% for *N. gonorrhoeae*.

Potential contributors included the uneven geographical distribution of the evidence, differences in recruitment settings and population composition, and variation in diagnostic methods and specimen types, as described in Section 3.2 and Section 3.3. Brazil contributed most of the available evidence, whereas several countries were represented by only one study and others had no eligible evidence. The included populations also ranged from general or routine-care groups to symptomatic, pregnant, screening-based, and selected or high-risk populations.

The exploratory subgroup analyses did not identify a consistent single source of heterogeneity under random-effects models. Under the original population-type classification presented in Table S4, with the corresponding subgroup analyses shown in Figures S1–S8, the only statistically significant population-type difference was observed for *G. vaginalis*, although this comparison included only three studies and one subgroup was represented by a single study. In the additional three-category population-setting analyses presented in Figures S12–S16, statistically significant differences were observed for *Candida* spp. and *G. vaginalis*, whereas no statistically significant differences were identified for *C. trachomatis*, *N. gonorrhoeae*, or *T. vaginalis*. Because the two sets of analyses used different population-classification frameworks, their results should be regarded as complementary exploratory assessments rather than directly interchangeable subgroup tests.

Accordingly, the pooled prevalence estimates should be interpreted as broad regional summaries across heterogeneous study contexts rather than as directly transferable prevalence parameters for individual countries, healthcare settings, or female populations.

### 3.7. Assessment of Reporting Bias and Small-Study Effects

Assessment of reporting bias and small-study effects was restricted to pathogen-specific meta-analyses based on studies eligible for the main pooled prevalence synthesis. Funnel plots and Egger regression tests were performed for *C. trachomatis* (33 studies), *T. vaginalis* (13 studies), and *N. gonorrhoeae* (10 studies), because these syntheses met the predefined threshold of at least 10 studies. Egger regression indicated evidence of funnel plot asymmetry for *C. trachomatis* (*p* = 0.0007) and *T. vaginalis* (*p* = 0.0071), whereas no statistically significant evidence of asymmetry was identified for *N. gonorrhoeae* (*p* = 0.5031). Formal assessment was not performed for *Candida* spp. or *G. vaginalis* because only six and three studies, respectively, contributed to these syntheses. These findings should be interpreted cautiously because, in prevalence meta-analyses, apparent asymmetry may reflect substantial between-study heterogeneity, sparse data, unequal study sizes, or variation in population and study design rather than publication bias alone (Table S7; Figures S9–S11).

### 3.8. Certainty of Evidence

Across all five pathogen-specific prevalence outcomes, the overall certainty of evidence was judged to be very low using an adapted domain-based assessment for prevalence studies. This reflected the combination of moderate-to-serious concerns regarding study risk of bias, very serious inconsistency due to the substantial heterogeneity observed across all main meta-analyses, important indirectness arising from heterogeneous populations and diagnostic approaches, and imprecision in several pathogen-specific syntheses. In addition, possible small-study effects were suspected for some outcomes when formally assessable. Full certainty judgments and justifications are presented in Table S8.

### 3.9. Supplementary Subgroup Analyses

The original exploratory subgroup meta-analyses were conducted according to diagnostic method and the harmonized population-type classification presented in Table S4, when sufficient data and comparable subgroup strata were available. Formal interaction between subgroup categories was evaluated using the between-subgroup Cochran’s Q test, with random-effects results prioritized for interpretation. No statistically significant subgroup separation by diagnostic method was identified for *C. trachomatis* (*Q*_between_ = 0.08, df = 1, *p* = 0.7741), *T. vaginalis* (*p* = 0.7610), or *G. vaginalis* (*p* = 0.3136).

Under the original harmonized population-type classification, subgroup differences were not statistically significant for *Candida* spp. (*p* = 0.6088), *C. trachomatis* (*p* = 0.0542), *T. vaginalis* (*p* = 0.3422), or *N. gonorrhoeae* (*p* = 0.4605). A statistically significant difference was observed for *G. vaginalis* (*p* < 0.0001); however, this analysis included only three studies and one subgroup was represented by a single study. These original diagnostic-method and population-type analyses are presented in Figures S1–S8.

To further examine population heterogeneity across the included studies, additional post hoc exploratory subgroup analyses were conducted using three broader population-setting categories: selected/high-risk, urban general, and remote/rural populations. For *Candida* spp., the random-effects pooled prevalence was 25% (95% CI: 21–30%) in selected/high-risk populations and 10% (95% CI: 3–29%) in urban general populations, while the only remote/rural study reported a prevalence of 3% (95% CI: 1–6%). The between-subgroup difference was statistically significant (*p* < 0.0001), although the comparison was based on a small number of studies and one subgroup was represented by a single study. For *C. trachomatis*, pooled prevalence was 5% (95% CI: 2–11%) in selected/high-risk populations, 8% (95% CI: 7–10%) in urban general populations, and 6% (95% CI: 3–13%) in remote/rural populations, with no evidence of a subgroup difference under the random-effects model (*p* = 0.3516).

For *G. vaginalis*, pooled prevalence was 42% (95% CI: 38–46%) in urban general populations, whereas the single remote/rural study reported 9% (95% CI: 5–13%). The subgroup-difference test was statistically significant (*p* < 0.0001), but this comparison included only three studies and should therefore be interpreted cautiously. For *N. gonorrhoeae*, pooled prevalence was 5% (95% CI: 2–15%) in selected/high-risk populations, 2% (95% CI: 0–20%) in urban general populations, and 3% (95% CI: 1–7%) in the only remote/rural study, with no significant subgroup difference (*p* = 0.6896). For *T. vaginalis*, pooled prevalence was 6% (95% CI: 4–10%) in selected/high-risk populations, 5% (95% CI: 2–11%) in urban general populations, and 14% (95% CI: 3–47%) in remote/rural populations; however, the between-subgroup difference was not statistically significant (*p* = 0.4652). These analyses are presented in Figures S12–S16.

Considerable heterogeneity persisted within several subgroup strata, and some categories contained only one or two studies. The original population-type analyses and the additional three-category population-setting analyses also yielded partially different patterns because they applied distinct but complementary study-classification frameworks. The original analyses evaluated the previously harmonized population-type categories, whereas the additional analyses incorporated both epidemiological risk and urban–rural setting. Accordingly, differences in subgroup estimates and interaction-test results should not be interpreted as inconsistencies or evidence of independent population-setting effects, but as exploratory findings arising from complementary classifications of a heterogeneous evidence base.

### 3.10. Exploratory Assessment of Reported Demographic and Behavioral Covariates

Exploratory cross-study analyses of reported demographic and behavioral covariates were undertaken as a descriptive, hypothesis-generating component of the review. Using the binary coding framework summarized in Table S5, 18 reported covariates were compared across the five evaluated pathogens, yielding 90 pathogen–covariate comparisons derived from 46 studies. Most comparisons did not show reproducible cross-study patterns, and no association remained statistically significant after Benjamini–Hochberg correction for multiple testing. Given the heterogeneous and incomplete reporting of covariates across studies, these findings should be interpreted strictly as descriptive and not as a robust synthesis of pathogen-specific risk factors (Table S6).

## 4. Discussion

This systematic review and meta-analysis provides, to our knowledge, the most comprehensive regional synthesis currently available on the prevalence of five clinically relevant genital microorganisms among women in South America during 2014–2024. Across 51 studies conducted in eight South American countries and French Guiana, pooled prevalence estimates were highest for *G. vaginalis*, followed by *Candida* spp., *C. trachomatis*, *T. vaginalis*, and *N. gonorrhoeae.* The main contribution of this review lies not in proposing a single definitive prevalence value for each microorganism, but in documenting the broad regional burden, the marked heterogeneity across study settings, and the uneven geographical distribution of available evidence. In this context, differences between crude and pooled estimates should be interpreted as reflecting variation in study size, study populations, and methodological diversity rather than as simple numerical inconsistencies. Accordingly, the pathogen-specific prevalence synthesis constitutes the core finding of the review, whereas subgroup and covariate analyses should be interpreted as secondary exploratory components that contextualize, but do not redefine, the main results.

From a public health perspective, these findings indicate that microorganisms associated with vaginal dysbiosis and candidal overgrowth account for a substantial part of the microbiological burden captured across the female populations represented in the regional literature, while treatable sexually transmitted infections remain important contributors to women’s reproductive health. This interpretation is consistent with current international guidance, which recognizes bacterial vaginosis, vulvovaginal candidiasis, and trichomoniasis as important causes of lower genital tract symptoms, while also emphasizing that cervical infections caused by *C. trachomatis* or *N. gonorrhoeae* may occur with or without overt vaginal discharge and are less reliably predicted by symptoms alone [66,67,68]. This pattern is clinically meaningful because not all detected microorganisms have the same mechanistic relationship with specific genital manifestations.

This interpretation is supported by studies included in the review. In pregnant women from southern Brazil, Yeganeh et al. [61] documented a measurable burden of laboratory-confirmed STIs in antenatal-care settings, while Melo et al. [43] reported discordance between clinical and laboratory diagnoses among Chilean women. These findings reinforce that cervical STIs such as *C. trachomatis* and *N. gonorrhoeae* may occur with or without clinically evident discharge and may be missed when management relies exclusively on symptoms. In contrast, *G. vaginalis* and *Candida* spp. are more directly associated with dysbiosis-related and vulvovaginal presentations. Therefore, the pooled estimates should not be interpreted as an etiologic ranking of a single genital syndrome, but rather as the regional distribution of five clinically relevant microorganisms with distinct anatomical, clinical, and epidemiological profiles [43,61,66,67,68].

The geographical distribution of the included evidence reveals marked asymmetry in regional research coverage. Although eligible studies were identified from eight South American countries and French Guiana, no eligible studies were identified from Paraguay, Bolivia, or Uruguay, and Brazil alone accounted for 31 of the 51 included studies. Figure 2 describes only the relative composition of microorganism-positive detections reported by the studies contributing data within each country. Because the contributing studies differed in the microorganisms tested, populations sampled, diagnostic approaches, and sample sizes, the displayed proportions cannot be used to compare population-level prevalence, national burden, or relative infection risk across countries. The figure should therefore be interpreted as an evidence-mapping visualization rather than an epidemiological comparison between national populations. This uneven distribution of evidence is consistent with the broader regional literature indicating that reliable population-based STI prevalence data remain limited across Latin America and the Caribbean and that important variation in *C. trachomatis* and *N. gonorrhoeae* estimates persists across countries and study populations [8,68].

This evidence gap may also reflect broader structural differences in research capacity and health-system organization across the region. Potential contributors include variation in public-health and research priorities, the integration of individual STIs into national surveillance programs, access to molecular diagnostic platforms, availability of trained laboratory personnel, and coordination between clinical services, reference laboratories, universities, and surveillance authorities [9,10,11,68]. Geographic dispersion, rural and remote populations, fragmented referral networks, and unequal access to sexual and reproductive health services may further limit representative specimen collection and the implementation of population-based prevalence studies [9,10]. In addition, locally generated surveillance or research findings may not always be disseminated through readily indexed peer-reviewed publications. The absence of eligible publications from Bolivia, Paraguay, and Uruguay should therefore be interpreted as an evidence gap within the scope and eligibility criteria of this review, rather than as evidence that diagnostic or surveillance activities are absent in those countries.

The regional landscape captured by this review is also shaped by highly diverse study populations, including pregnant women, women attending primary care or cervical screening services, indigenous and quilombola communities, women from remote Amazonian areas, adolescents and young adults, university populations, female sex workers, and women living with HIV [22,23,24,25,30,34,36,37,43,47,49,52,53,54,55,56,62,63]. Taken together, these differences in evidence availability, research infrastructure, diagnostic access, and sampled populations indicate that the pooled estimates reported here should be interpreted as summary measures across heterogeneous female populations rather than as directly comparable country-level prevalence parameters [8,68].

The predominance of *G. vaginalis* observed in this review is consistent with the recognized importance of bacterial vaginosis-related dysbiosis in women presenting with genital symptoms [66,69,70,71]. At the same time, detection of *G. vaginalis* should not be considered synonymous with a clinical diagnosis of bacterial vaginosis, particularly because the included studies used heterogeneous diagnostic approaches, including molecular assays, Gram staining-based methods, and broader microbiota-oriented assessments [69,70,71]. The difference between the crude and pooled estimates for *G. vaginalis* reflects the influence of study weighting and the marked variability across the three contributing studies, as also indicated by the very high between-study heterogeneity. Similarly, *Candida* spp. showed a substantial pooled prevalence, reinforcing their importance as frequent contributors to vulvovaginal symptoms in clinical practice [66,67,72]. The observed variability across studies is biologically plausible and likely reflects differences in pregnancy status, antibiotic exposure, metabolic and hormonal conditions, and laboratory methodology, including the contrast between microscopy, culture, and molecular detection [72,73,74]. The difference between the crude prevalence of 13.46% and the random-effects pooled estimate of 12% reflects study weighting and substantial variability across the six contributing studies.

The predominance of dysbiosis-related and candidal findings is also consistent with data from individual studies included in the review. In Ecuador, Salinas et al. reported frequent detection of vaginal microorganisms associated with dysbiosis and infection, showing that *Candida*-related findings formed part of a broader polymicrobial landscape rather than an isolated phenomenon [59]. In asymptomatic pregnant women from Colombia, Gómez-Rodríguez et al. also identified microbiological abnormalities relevant to bacterial vaginosis, candidiasis, and trichomoniasis despite the absence of overt symptoms [41]. In Argentina, Mucci et al. further documented vulvovaginal candidiasis and the distribution of clinically relevant *Candida* species in pregnant women, reinforcing that candidal detection represents a recurrent component of the lower genital tract microbiological burden in this population [46]. Together, these included studies support the interpretation that vaginal dysbiosis and *Candida* spp. represent recurrent components of the lower genital tract microbiological burden in South American women [41,46,59].

In contrast, the pooled prevalence of *C. trachomatis* indicates that this cervical sexually transmitted infection remains epidemiologically relevant among South American women, including in settings where symptoms may be absent, mild, or not clearly attributable to cervical infection [75,76]. The modest difference between pooled and crude prevalence estimates suggests that the regional summary measure was shaped by between-study heterogeneity, study weighting, and variation in population composition and diagnostic approach across the included studies. This interpretation is consistent with the composition of the available evidence, which encompassed younger women, pregnant women, remote Amazonian populations, and female students, all of which may contribute distinct epidemiological risk structures. From a clinical perspective, these findings reinforce the need for screening and diagnostic strategies that extend beyond symptom-based approaches, given the established links between untreated chlamydial infection, pelvic inflammatory disease, infertility, and adverse pregnancy outcomes [75,76].

The pooled prevalence of *T. vaginalis* further supports its persistence as an underrecognized sexually transmitted infection in the region. The modest difference between pooled and crude prevalence estimates suggests that the regional summary measure may have been influenced by between-study heterogeneity, the concentration of infection in selected populations, and differential detection across diagnostic methods. This interpretation is consistent with evidence showing that molecular assays outperform wet mount microscopy and some conventional diagnostic approaches for trichomoniasis [67,77,78]. Accordingly, part of the apparent variability in *T. vaginalis* detection across South America may reflect methodological differences rather than epidemiological variation alone. Our estimate is closely aligned with the findings of Tian et al. [79], whose systematic review and meta-analysis of 425 studies reported a global *T. vaginalis* prevalence of 8% (95% CI: 7–10%), compared with 6% (95% CI: 3–10%) in the present South American synthesis [79]. Tian et al. also found higher odds of *T. vaginalis* infection among individuals with other sexually transmitted infections, including HIV, herpes simplex virus, and *C. trachomatis* infection (pooled OR: 2.01; 95% CI: 1.48–2.72), as well as among groups characterized by smoking, drug use, or non-use of condoms (pooled OR: 1.67; 95% CI: 1.39–2.00) [79]. Socioeconomic factors, including unmarried status, low income, and unstable employment, were also associated with greater infection risk (pooled OR: 1.36; 95% CI: 1.10–1.66) [79]. These global findings support the epidemiological plausibility of the regional estimate obtained in our review and reinforce the importance of considering coinfections, behavioral exposures, and social vulnerability when interpreting variation in *T. vaginalis* positivity across study populations.

The comparatively low pooled prevalence of *N. gonorrhoeae* should not be interpreted as evidence of limited clinical importance. Even at low prevalence, gonococcal infection remains highly relevant because of its reproductive consequences and the growing challenge of antimicrobial resistance [68,80]. The difference between the random-effects pooled prevalence of 3.2% and the crude prevalence of 0.81% reflects the highly unequal study sizes and the extreme between-study heterogeneity observed for *N. gonorrhoeae* (*I*^2^ = 99.2%). The crude estimate was calculated by aggregating all positive cases and tested women and was therefore dominated by the largest study, which reported very low positivity. In contrast, the random-effects model assigned relatively greater influence to smaller studies conducted in clinical, antenatal, HIV-related, homeless, or other selected populations in which higher positivity was observed. Consequently, the pooled estimate was shifted upward despite the low overall proportion of positive cases. The wide 95% confidence interval of the random-effects estimate (0.8–12.6%) further indicates that the summary value is imprecise and should not be interpreted as a uniform population-level prevalence across South America.

Several included studies help contextualize the pooled estimates for classical STIs. *C. trachomatis* was repeatedly documented across diverse South American settings, including antenatal care, university populations, adolescents, women attending cytology or primary care services, and women living in geographically remote communities [19,22,29,36,47,49,50,52,55,56,62,63]. This pattern supports the interpretation that chlamydial infection remains broadly distributed beyond classical STI clinic populations. In addition, studies from Brazil, Colombia, and Chile reported detectable STI burdens in women who were asymptomatic or only partially symptomatic, again illustrating the limitations of syndromic care [33,49,55,62]. For *T. vaginalis*, studies using molecular assays or NAAT-based approaches suggested that reliance on conventional methods alone may underestimate infection burden [18,26,33,65]. Although *N. gonorrhoeae* showed the lowest pooled prevalence, its detection across antenatal, primary care, HIV-related, and STI-oriented settings supports continued surveillance, particularly in view of its public health relevance and antimicrobial resistance concerns [20,33,40,53,54,61,64].

A major methodological feature of this review was the marked variability in diagnostic approaches and specimen sources across the included studies. Although molecular methods predominated, conventional techniques such as culture, microscopy, Gram staining, and clinical diagnosis were also used, sometimes in combination, and specimen types ranged from vaginal or cervicovaginal swabs to cervical or endocervical samples, urine, and mixed sources. This variability likely influenced pathogen detection and plausibly contributed to the very high between-study heterogeneity observed across all pathogen-specific meta-analyses. In this field, such heterogeneity is not unexpected, because case definitions, sampling strategies, specimen selection, and laboratory capacity differ substantially across settings. Accordingly, the pooled prevalence estimates reported here should be interpreted as regional approximations across heterogeneous clinical and epidemiological contexts rather than as fixed epidemiological constants [66,67,68,69,70,71,74,81,82].

Two complementary population-based subgroup frameworks were evaluated. The original harmonized population-type analyses were retained as the initially defined exploratory assessments, while an additional three-category population-setting framework was applied to distinguish selected/high-risk, urban general, and remote/rural populations. Because these frameworks classified studies according to related but non-identical criteria, differences in their subgroup estimates and interaction-test results were expected and should not be interpreted as contradictory.

The exploratory population-setting analyses showed that prevalence patterns were not uniform across microorganisms. For *C. trachomatis*, *N. gonorrhoeae*, and *T. vaginalis*, the random-effects subgroup tests did not demonstrate statistically significant differences among selected/high-risk, urban general, and remote/rural populations. Although the point estimate for *N. gonorrhoeae* was higher in selected/high-risk populations than in urban general populations (5% vs. 2%), the corresponding confidence intervals were wide and overlapped substantially. Likewise, the apparently higher prevalence of *T. vaginalis* in remote/rural populations (14%) compared with selected/high-risk (6%) and urban general populations (5%) was imprecise and was not supported by a statistically significant subgroup difference.

Statistically significant subgroup differences were observed for *Candida* spp. and *G. vaginalis*. *Candida* spp. prevalence was higher in selected/high-risk populations than in urban general populations (25% vs. 10%), whereas the only remote/rural study reported 3%. For *G. vaginalis*, urban general populations showed a pooled prevalence of 42%, compared with 9% in the single remote/rural study. However, these findings should not be interpreted as definitive urban–rural or risk-group gradients because the comparisons were based on few studies, some subgroups contained only one study, and population classification could not fully separate clinical selection, pregnancy, symptoms, behavioral risk, and geographic setting.

Substantial heterogeneity persisted within several population strata, indicating that broad population-setting categories alone did not account for the variability across studies. Differences in age, pregnancy status, symptom profile, HIV status, sexual behavior, healthcare access, specimen type, diagnostic method, and local epidemiology may also have contributed. These subgroup findings should therefore be interpreted as descriptive and hypothesis-generating rather than as evidence of an independent population-setting effect.

The exploratory cross-study assessment of demographic and behavioral covariates yielded only limited descriptive signals, and none remained statistically significant after correction for multiple testing. These findings should not be interpreted as evidence for the absence of epidemiological relationships between specific covariates and pathogen detection. Rather, they reflect the fact that the available evidence was sparse, inconsistently reported, and not generated within a standardized analytical framework designed for cross-study association testing. Because most included studies were designed primarily to estimate prevalence, the present review can offer only a descriptive and hypothesis-generating overview of reported covariate patterns, not a definitive synthesis of risk factors.

Host-related conditions and concurrent genital infections may also have influenced the study-specific positivity estimates. Several included studies evaluated pregnant women, women living with HIV, or populations in which multiple sexually transmitted or genital microorganisms were investigated simultaneously [19,21,22,24,32,33,36,37,40,41,44,46,53,54,55,56,57,58,61,63]. Pregnancy and HIV infection may modify susceptibility to genital infection or the persistence and clinical expression of detected microorganisms, while interactions within the vaginal microbiota may influence colonization, dysbiosis, and the coexistence of sexually transmitted pathogens [3,6,7,12,72]. However, the included studies reported comorbid conditions and coinfections inconsistently and frequently lacked uniform clinical definitions, pathogen-specific numerators and denominators, appropriate comparison groups, or adjusted effect estimates. Consequently, their relationship with microorganism positivity could not be synthesized quantitatively, and the available observations should be interpreted as descriptive rather than as evidence of independent effects.

Several limitations should be considered when interpreting the findings of this review. Between-study heterogeneity was consistently considerable, with *I*^2^ values ranging from 91.5% to 99.2%, limiting the precision of regional pooled estimates. Substantial variation in laboratory methods and specimen types may also have introduced differential detection and misclassification across studies. Because several studies used clinical, convenience, screening-based, or otherwise-selected samples rather than population-representative sampling frameworks, their study-specific estimates may more appropriately reflect positivity within the sampled populations than population-level prevalence. Selective microorganism testing, incomplete reporting of covariates, and the absence of eligible studies from several South American countries may likewise have biased comparability and limited the scope of subgroup interpretation. The additional three-category population-setting classification was based on study-level descriptions and may have combined populations with different clinical, behavioral, and social characteristics. Moreover, because this framework differed from the original harmonized population-type classification, the two sets of subgroup analyses should not be compared as if they represented identical contrasts. Several subgroup strata also contained only one or two studies, limiting the reliability of pooled estimates and formal tests of subgroup differences. Similarly, inconsistent reporting of comorbidities and coinfections, together with the absence of comparable pathogen-stratified denominators and effect estimates, prevented a formal meta-analysis of their relationship with microorganism positivity. The lack of eligible publications from some countries may reflect differences in research and surveillance capacity, diagnostic access, dissemination practices, or database indexing rather than a complete absence of locally generated information. Formal assessment of reporting bias and small-study effects was not performed for *G. vaginalis* and *Candida* spp. because only three and six studies, respectively, contributed to these pathogen-specific syntheses. Consequently, publication bias or other small-study effects cannot be ruled out for these outcomes. Although backward citation searching of the reference lists of eligible studies and relevant reviews was performed, formal forward citation tracking of the included articles was not conducted. Consequently, additional eligible regional prevalence reports may have been missed, particularly recently published studies or records with limited indexing or visibility in the databases searched. These issues support interpreting the pooled estimates as regional approximations rather than precise epidemiological parameters. Formal sensitivity analyses were not performed, and the robustness of pooled estimates should therefore be interpreted in light of the substantial between-study heterogeneity and the exploratory nature of the secondary analyses. The demographic and behavioral covariate analyses were based on unadjusted cross-study comparisons and could not account consistently for confounding, effect modification, or differences in population composition. Moreover, the included studies did not use uniform risk-factor definitions, reference categories, exposure measurements, or association metrics, which prevented a harmonized adjusted synthesis. Consequently, these exploratory findings should not be interpreted as evidence of causal risk-factor relationships or as estimates of independent associations; they should be regarded solely as descriptive and hypothesis-generating.

Despite these limitations, this review provides a decade-spanning regional synthesis and identifies several priorities for translating the available evidence into public-health action. First, countries should progressively adopt standardized, laboratory-supported, multi-pathogen diagnostic algorithms that distinguish sexually transmitted pathogens from dysbiosis-related conditions and that are adapted to local laboratory capacity. In remote Amazonian, rural, and geographically dispersed populations, decentralized specimen collection, validated self-sampling strategies, and point-of-care or near-point-of-care molecular platforms could improve access to diagnosis while reducing dependence on distant reference laboratories.

Second, the frequent detection of infections among asymptomatic women, pregnant women, adolescents, young adults, and women attending primary-care or cervical-screening services supports the evaluation of targeted screening programs rather than symptom-based testing alone. In countries such as Chile and Colombia, where the included evidence identified infections among asymptomatic women and users of primary-care or cervical-screening services, pilot screening strategies could initially focus on younger women, pregnant women, and other locally defined higher-risk groups before considering broader national implementation. Such programs should include referral pathways, partner management, treatment access, and periodic assessment of cost-effectiveness and feasibility.

Third, countries with limited or absent eligible prevalence evidence should be prioritized in regional research and surveillance initiatives. A South American laboratory and surveillance network could harmonize case definitions, specimen collection, molecular testing panels, quality assurance, and minimum reporting datasets. Cross-border collaboration would be particularly relevant in the Southern Cone and other areas with substantial population mobility, while regional sharing of gonococcal antimicrobial-susceptibility data could strengthen the early detection of resistant strains. These actions should be accompanied by sustained investment in laboratory personnel, reference capacity, interoperable surveillance systems, and the inclusion of rural, indigenous, migrant, and other underserved female populations [66,67,68,74,80,81,82].

## Figures and Tables

**Table 1:** Crude prevalence (*n*/*N*) and random-effects pooled prevalence of five clinically relevant genital microorganisms among women in eight South American countries and French Guiana, 2014–2024. Data are presented as percentages (%) with the corresponding number of positive cases and total number of tested individuals (*n*/*N*). Pooled prevalence estimates were calculated using random-effects meta-analysis models. Crude prevalence was calculated directly from the observed number of positive cases and total tested individuals (*n*/*N*), without meta-analytic weighting. The 95% confidence intervals (95% CIs) for crude prevalence were calculated assuming a binomial distribution. Forest plots corresponding to pooled prevalence estimates are provided in Figure 4, Figure 5, Figure 6, Figure 7 and Figure 8. Differences between crude and pooled prevalence values reflect the influence of study weighting and between-study heterogeneity.

Pathogen	*n*/*N*	Random-Effects Pooled Prevalence (%)	95% CI	Crude Prevalence (%)	95% CI
*G. vaginalis*	322/957	28.00	13.00–49.00	33.65	30.66–36.74
*Candida* spp.	199/1479	12.00	6.00–22.00	13.46	11.76–15.30
*C. trachomatis*	1151/14,635	7.00	6.00–9.00	7.86	7.43–8.31
*T. vaginalis*	352/5146	6.00	3.00–10.00	6.84	6.17–7.56
*N. gonorrhoeae*	315/39,033	3.20	0.80–12.60	0.81	0.72–0.90

Abbreviations: CI, confidence interval; *n*, number of positive cases; *N*, total number of individuals tested.

## Data Availability

All data underlying the findings of this study, including the extracted study-level dataset provided as Data S1 and the R code used for the main pathogen-specific meta-analyses, subgroup analyses, and funnel plots assessing reporting bias, are available in the Zenodo repository at DOI: 10.5281/zenodo.21810943. Additional supporting materials are provided with the manuscript.

## Supplementary Materials

The following supporting information can be downloaded at: https://www.mdpi.com/article/10.3390/pathogens15090888/s1, Checklist S1. PRISMA 2020 checklist; Data S1. Extracted study-level dataset underlying the main prevalence meta-analyses, subgroup analyses, and exploratory cross-study comparisons. Figure S1. Subgroup analysis forest plot of *Candida* spp. prevalence by population type. Figure S2. Subgroup analysis forest plot of *Chlamydia trachomatis* prevalence by diagnostic method. Figure S3. Subgroup analysis forest plot of *Chlamydia trachomatis* prevalence by population type. Figure S4. Subgroup analysis forest plot of *Gardnerella vaginalis* prevalence by diagnostic method. Figure S5. Subgroup analysis forest plot of *Gardnerella vaginalis* prevalence by population type. Figure S6. Subgroup analysis forest plot of *Neisseria gonorrhoeae* prevalence by population type. Figure S7. Subgroup analysis forest plot of *Trichomonas vaginalis* prevalence by diagnostic method. Figure S8. Subgroup analysis forest plot of *Trichomonas vaginalis* prevalence by population type. Figure S9. Funnel plot for *Chlamydia trachomatis* prevalence across studies included in the main pooled prevalence meta-analysis. Figure S10. Funnel plot for *Trichomonas vaginalis* prevalence across studies included in the main pooled prevalence meta-analysis. Figure S11. Funnel plot for *Neisseria gonorrhoeae* prevalence across studies included in the main pooled prevalence meta-analysis. Figure S12. Subgroup analysis forest plot of *Candida* spp. prevalence by selected/high-risk, urban general, and remote/rural population setting. Figure S13. Subgroup analysis forest plot of *Chlamydia trachomatis* prevalence by selected/high-risk, urban general, and remote/rural population setting. Figure S14. Subgroup analysis forest plot of *Gardnerella vaginalis* prevalence by urban general and remote/rural population setting. Figure S15. Subgroup analysis forest plot of *Neisseria gonorrhoeae* prevalence by selected/high-risk, urban general, and remote/rural population setting. Figure S16. Subgroup analysis forest plot of *Trichomonas vaginalis* prevalence by selected/high-risk, urban general, and remote/rural population setting. Table S1. Full database-specific search strategies used for the systematic literature search. Table S2. Data extraction matrix summarizing the main characteristics of the studies included. Table S3. JBI critical appraisal, overall risk-of-bias classification, and eligibility for the main pooled prevalence meta-analysis. Table S4. Study-level classification used for subgroup analyses by diagnostic method and population type. Table S5. Harmonized binary coding of reported demographic and behavioral covariates across included studies. Table S6. Exploratory cross-study comparisons of reported covariates and pathogen detection, including odds ratios, 95% confidence intervals, and adjusted p values. Table S7. Assessment of reporting bias and small-study effects across pathogen-specific meta-analyses. Table S8. Certainty of evidence by pathogen using an adapted GRADE-style assessment for prevalence outcomes. Table S9. Reports sought for retrieval but not obtained after reasonable full-text retrieval efforts, including available bibliographic details and retrieval approaches.
